# Statistical results on restorative dentistry experiments: effect of the interaction between main variables

**DOI:** 10.1590/S1678-77572010000300010

**Published:** 2010

**Authors:** Andrea Nóbrega CAVALCANTI, Giselle Maria MARCHI, Gláucia Maria Bovi AMBROSANO

**Affiliations:** 1 DDS, MSc, PhD, Associate Professor, Department of Oral Rehabilitation, School of Dentistry, School of Medicine and Public Health of Bahia (EBMSP), Salvador, BA, Brazil.; 2 DDS, MSc, PhD, Associate Professor, Department of Restorative Dentistry, Piracicaba Dental School, State University of Campinas, Piracicaba, SP, Brazil.; 3 MSc, PhD, Full Professor, Department of Community Dentistry and Biostatistics, Piracicaba Dental School, State University of Campinas, Piracicaba, SP, Brazil.

**Keywords:** Biostatistics, Dental research, Analysis of variance

## Abstract

Statistical analysis interpretation is a critical field in scientific research. When there is more than one main variable being studied in a research, the effect of the interaction between those variables is fundamental on experiments discussion. However, some doubts can occur when the p-value of the interaction is greater than the significance level.

**Objective:**

To determine the most adequate interpretation for factorial experiments with p-values of the interaction nearly higher than the significance level.

**Materials and methods:**

The p-values of the interactions found in two restorative dentistry experiments (0.053 and 0.068) were interpreted in two distinct ways: considering the interaction as not significant and as significant.

**Results:**

Different findings were observed between the two analyses, and studies results became more coherent when the significant interaction was used.

**Conclusion:**

The p-value of the interaction between main variables must be analyzed with caution because it can change the outcomes of research studies. Researchers are strongly advised to interpret carefully the results of their statistical analysis in order to discuss the findings of their experiments properly.

## INTRODUCTION

Factorial experiments are those in which more than one main factor is studied. This type of statistical design is frequently employed on dental research^2,3,8,9,11,13,15^. The important feature behind this experimental design is that the effects of a number of different main variables are investigated simultaneously, and all associations between the different variables are considered in the analysis. In the case of an experiment with two main variables, both presenting two levels of variation, the experiment is described as a 2x2 factorial experiment, and so on^4^.

The factorial experiment demonstrates advantages over other statistical designs^7^. It enables efficient simultaneous investigation of two or more interventions, including all participants in their analyses. Also, in a factorial design it is possible to consider the benefits of receiving all interventions together and the isolated effects of each intervention^7,10,12^.

The p-value indicates the probability of seeing the observed difference, or greater, just by chance if the null hypothesis is true. Values close to 0 indicate that the observed difference is unlikely to be due to chance, whereas a p-value close to 1 suggests that there is no difference between groups other than that due to random variation^16^. In a factorial design, data calculations establish one p-value for each involved factor and another for the interaction between them.

A significant interaction between two factors indicates that the effect of one variable depends on the levels of the second variable^14^. As a general rule, the interpretation of the p-value of the interaction should be done first, and if this p-value is not significant, then the main effects could be examined separately^14^. However, researchers sometimes find the results of a factorial experiment difficult to interpret, especially when there are multiple main variables included in the experimental design. In addition, there is always a controversy on how to interpret the p-value of the interaction, when it is nearly greater than the significance level (i.e. α=5% / α=0.05). In order to determine the most adequate interpretation for factorial experiments, the aim of the present study was to analyze p-values from the interaction nearly greater than 0.05 in two distinct ways: considering the interaction as not significant and as significant. The tested hypothesis was that considering such p-values as significant induces more realistic data interpretation.

## MATERIAL AND METHODS

Two restorative dentistry experiments with the p-value from the interaction nearly greater than the significance level (α=0.05) were selected. Two approaches were investigated: assuming no interaction, and presupposing a significant interaction.

### Experimental design

In the first study, 60 restorations on bovine teeth were used as experimental units. The main effects tested were: bonding system [3 levels of variation: Single Bond (3M ESPE, St. Paul, MN, USA), Clearfil Se Bond (Kuraray, Tokyo, Japan), OptiBond Solo Plus (Kerr Corp., Orange, CA, USA)] and aging procedure (2 levels of variation: mechanical and mechanical-thermal). This study represented a 3 x 2 factorial design. The dependent variable was the tensile bond strength (TBS) in MPa.

The experimental units of the second study were 60 composite resin blocks. The main effects were: composite resin (3 levels of variation: hybrid, microhybrid, microfilled) and curing time (2 levels of variation – 20 s and 60 s) – a 3x2 factorial design. The dependent variable was the Knoop hardness number (KHN).

Results from both experiments were evaluated for statistical significance using two-way ANOVA and Tukey’s test for multiple comparisons. All statistical analyses were conducted using SAS 8.0 software (SAS Institute, Cary, NC, USA).

## RESULTS

In the TBS experiment, the p-value of the interaction was 0.053. When this interaction was considered not significant, only the factor bonding system presented a statistical significance, and the Clearfil SE Bond system presented bond strength means significantly lower than the other systems. even though the effect of the aging procedure on restorations bond strength seemed clear when Single Bond means were observed, this effect was not statistically significant (Table 1).

**Table 1:** Mean (standard deviation) obtained in experiment 1. Statistical analysis considering the p-value of the interaction (p=0.053) as not significant

**Aging Procedure**		**Bonding systems**		
	**Single Bond**	** Clearfil SE Bond**	**OptiBond Solo**	
				
Mechanical	32.61 (6.84)	24.21 (6.78)	27.63 (4.63)	a
Mechanical + Thermal	25.86 (7.39)	20.08 (5.39)	25.87 (5.36)	a
	A	B	A	

Different letters represent statistically significant differences (Two-way ANOVA / Tukey test, α=5%). Uppercase letters compare adhesive systems and lowercase letters compare aging procedures.

On the other hand, results changed considerably when this interaction was interpreted as significant. In this ultimate analysis, differences were observed between bonding systems and also between aging conditions (Table 2). The mean bond strength of Clearfil SE Bond system remained lower than those of the other systems. In addition, the effect of the aging procedure on Single Bond system bond strength that was not detected in the previous analysis was then considered as statistically significant.

**Table 2:** Mean (standard deviation) obtained in experiment 1. Statistical analysis considering the p-value of the interaction (p=0.053) as significant

**Aging Procedure**		**Bonding systems**		
	**Single Bond**	**Clearfil SE Bond**	**OptiBond Solo**	
				
Mechanical	32.61 (6.84) Aa	24.21 (6.78) Ab	27.63 (4.63) Aa	a
Mechanical + Thermal	25.86 (7.39) Ba	20.08 (5.39) Ab	25.87 (5.36) Aa	a

Different letters represent statistically significant differences (Two-way ANOVA / Tukey test, α=5%). Uppercase letters compare aging procedures and lowercase letters compare adhesive systems.

In the hardness experiment, the p-value of the interaction was 0.068. When this interaction was considered not significant, the hybrid composite presented significantly higher KHN compared to the other composites (Table 3). However, the levels of the factor curing time were statistically similar, meaning that composites presented the same behavior at the two curing times.

**Table 3:** Mean (standard deviation) obtained in experiment 2. Statistical analysis considering the p-value of the interaction (p=0.068) as not significant

**Curing Time**		**Composites**		
	**Hybrid**	** Microfilled**	**Microhybrid**	
				
20 s	49.82 (4.05)	47.48 (2.98)	47.91 (2.58)	a
60 s	53.84 (1.92)	47.23 (3.61)	48.37 (2.56)	a
	A	B	B	

Different letters represent statistically significant differences (Two-way ANOVA / Tukey test, α=5%). Uppercase letters compare composites and lowercase letters compare curing times.

In the second analysis, considering the interaction as significant; differences were observed among composite resins and between curing times (Table 4). When cured for 20 s, the hybrid and the microhybrid composites presented similar KHN, and both were different from the microfilled composite. When cured for 60 s, the hybrid composite presented significantly higher KHN compared to the other composites. The curing time was statistically significant for the hybrid composite, which presented higher mean after being cured for 60 s. The other composites were not affected by the curing time.

**Table 4:** Mean (standard deviation) obtained in experiment 2. Statistical analysis considering the p-value of the interaction (p=0.068) as not significant

**Curing Time**		**Composites**	
	**Hybrid**	** Microfilled**	**Microhybrid**
			
20 s	49.82 (4.05) Ba	47.48 (2.98) Ab	47.91 (2.58) Aa
60 s	53.84 (1.92) Aa	47.23 (3.61) Ab	48.37 (2.56) Ab

Different letters represent statistically significant differences (Two-way ANOVA / Tukey test, α=5%). Uppercase letters compare curing times and lowercase letters compare composites.

## DISCUSSION

Research validity depends on the proper analysis and interpretation of collected data. However, there are some controversial issues regarding statistical analysis that can dramatically change study’s conclusions, for example, the interpretation of the interaction between main variables. Usually, if a factorial design is selected for data assessment, researchers are probably expecting to find a dependent relationship between main variables. When this relationship is not an important issue, however, other statistical designs can be selected, for example, one-way ANOVA. This is why the p-value of the interaction becomes so important in a factorial analysis. Nevertheless, when this p-value is nearly greater than 0.05, researchers can doubt if this value can be considered statistically significant.

A common approach in the analysis of factorial trials is to assume p-values higher than the level of significance as not significant. Therefore, the interaction analysis is not adjusted for multiple testing. Even significant interactions are frequently ignored because some researchers seem to believe that the interpretation of the main effects separately could make data interpretation easier.

According to the findings of the present study, adjusting the interaction for multiple comparisons, even if the p-value is nearly greater than 0.05, provide considerably changes in experiments outcomes. In both experimental studies investigated, the interpretation of the significant interaction was advantageous for results discussion. Even though it is difficult to interpret the results from a factorial study with an influential interaction, the main advantage of such statistical design is the efficient and simultaneous investigation of two or more interventions^7^. In addition, this problem in interpreting results can be easily solved with continuous experience in similar analysis.

The sample size is an important issue for factorial designs when an interaction is being expected. If a study does not present an adequate power to detect an interaction, its sample size will have to be increased. With no increase in sample size, the interaction would need to be at least twice as large as the main effects to be detected with the same power^1,5-7^. Thus, researchers should appraise if a not significant interaction would present a different result if larger sample sizes were used.

Based on the results of this study, it can be suggested that the association between researchers and statisticians is fundamental for the establishment of the most adequate strategy to test experimental hypothesis. While researchers must decide which questions their experiments should answer, statisticians must determine the more adequate statistical method to achieve these objectives. In addition, considering the broad number of relevant information regarding data collection and analysis that can be brought by the p-value, researches should be strongly advised to indicate the exact value obtained rather than the discrimination of p-value greater or lower than 0.05.

## CONCLUSION

Within the limitations of this study, it may be concluded that analyses presented more reliable and realistic results when the p-value of interaction was considered as significant, even though it was slightly greater than the significance level. Thus, the hypothesis tested in this investigation was proven to be true.
